# Transplanted Human Amniotic Membrane-Derived Mesenchymal Stem Cells Ameliorate Carbon Tetrachloride-Induced Liver Cirrhosis in Mouse

**DOI:** 10.1371/journal.pone.0016789

**Published:** 2011-02-04

**Authors:** DingGuo Zhang, MinYue Jiang, DengShun Miao

**Affiliations:** The Research Center for Bone and Stem Cells, Nanjing Medical University, Nanjing, China; Health Canada, Canada

## Abstract

**Background:**

Human amniotic membrane-derived mesenchymal stem cells (hAMCs) have the potential to reduce heart and lung fibrosis, but whether could reduce liver fibrosis remains largely unknown.

**Methodology/Principal Findings:**

Hepatic cirrhosis model was established by infusion of CCl_4_ (1 ml/kg body weight twice a week for 8 weeks) in immunocompetent C57Bl/6J mice. hAMCs, isolated from term delivered placenta, were infused into the spleen at 4 weeks after mice were challenged with CCl_4_. Control mice received only saline infusion. Animals were sacrificed at 4 weeks post-transplantation. Blood analysis was performed to evaluate alanine aminotransferase (ALT) and aspartate aminotransferase (AST). Histological analysis of the livers for fibrosis, hepatic stellate cells activation, hepatocyte apoptosis, proliferation and senescence were performed. The donor cell engraftment was assessed using immunofluorescence and polymerase chain reaction. The areas of hepatic fibrosis were reduced (6.2%±2.1 vs. control 9.6%±1.7, p<0.05) and liver function parameters (ALT 539.6±545.1 U/dl, AST 589.7±342.8 U/dl,vs. control ALT 139.1±138.3 U/dl, p<0.05 and AST 212.3±110.7 U/dl, p<0.01) were markedly ameliorated in the hAMCs group compared to control group. The transplantation of hAMCs into liver-fibrotic mice suppressed activation of hepatic stellate cells, decreased hepatocyte apoptosis and promoted liver regeneration. More interesting, hepatocyte senescence was depressed significantly in hAMCs group compared to control group. Immunofluorescence and polymerase chain reaction revealed that hAMCs engraftment into host livers and expressed the hepatocyte-specific markers, human albumin and α-fetoproteinran.

**Conclusions/Significance:**

The transplantation of hAMCs significantly decreased the fibrosis formation and progression of CCl_4_-induced cirrhosis, providing a new approach for the treatment of fibrotic liver disease.

## Introduction

Liver cirrhosis is a common end-stage of a wide variety of chronic hepatic diseases caused by a variety of factors, such as viral infections, alcohol, drugs and chemical toxicity [1], [2], [3], [4]. It is often associated with the loss of functional liver cells, activation of hepatic stellate cells (HSC), the senescence of hepatocyte cells and accumulation of extracellular matrix, amongst other detrimental processes [3], [4], [5], [6]. Major advances have been made in the prevention, diagnosis, and treatment of liver cirrhosis, including the use of liver transplantation and artificial liver [7]. However, the number of patients suffering from liver disease is still increasing, and the availability of suitable donor livers is shortage. Morbidity and mortality from liver cirrhosis continue to be an enormous burden experienced by many individuals, with substantial economic cost [8], [9], [10]. Effective therapies to replace liver transplantation are clearly required.

Cell therapies are capable of complementing or replacing damaged liver cells. Enthusiasm for adult cell treatment for the injured liver has already reached the clinical setting, with physicians in several countries involved in clinical trials using mainly bone marrow-derived cells [11], [12], [13], [14], [15], [16]. However, not all trials get the positive results and these procedures even led to clinical harm in patients with established chronic liver disease [15]. Furthermore, harvesting the bone marrow to get cells is an invasive procedure for patients. Therefore, an ideal cell source to overcome the disadvantages of bone marrow-derived cells is clearly needed.

Recently, the multipotent differentiation ability of human amniotic membrane-derived mesenchymal stem cells (hAMCs) has been reported and these cells have attracted a lot of attention as a cell source for cell transplantation therapy [17], [18], [19]. Similar to bone marrow-derived cells, hAMCs have limited self-renewal ability, have low immunogenicity, and can be induced to various mesenchymal tissues and cells including those of hepatic lineage [19], [20]. Furthermore, unlike bone marrow-derived cells, they can be obtained non-invasively from the amnion membrane of term delivered placenta and easily cultured [18]. These characteristics are clear advantage of hAMCs, making them potentially superior to bone marrow-derived cells as a cell transplantation source. Recent research investigating the effects of hAMCs reported a decreased fibrosis area in infarcted myocardium [19] and a reduction in fibrosis in lungs of bleomycin injured mice [21]. However, whether hAMCs can be exploited following cell transplantation to reduce liver fibrosis remains largely unknown. In the present study, we transplanted hAMCs into immune competent C57Bl/6J mice with carbon tetrachloride (CCl_4_) induced hepatic cirrhosis and showed that hAMCs reduce HSC activation, protect hepatocyte from apoptosis, promote hepatocyte proliferation, and reduce hepatic fibrosis. More interesting, we confirmed hAMCs depressed hepatocyte senescence and differentiate into albumin-expressing or a-fetoprotein–expressing hepatocytes.

## Materials and Methods

### Animals

Four- to 6-week old C57Bl/6J mice were housed in a standard animal laboratory. They were kept at 23°C ∼25°C with a 12-hour light/dark cycle and allowed standard chow and water ad libitum until the time of the study. All animal experimental protocols were approved by the Animal Care and Use Committee of Nanjing Medical University (Approval ID 2009-08137) and were in compliance with Guidelines for the Care and Use of Laboratory Animals, as published by the National Academy Press (NIH Publication No. 85-23, revised 1996).

### hAMCs isolation and cultures

Human term placentas were obtained from healthy women with verbal consent (because the placentas were discarded) after caesarean section and processed immediately. The research procedure was approved by the Ethics Committee of the Nanjing Medical University. hAMCs were isolated according to the method previously described [19] with slight modification. Briefly, the amnion was manually separated from the chorion, washed extensively in phosphate-buffered saline (PBS) containing 100 U/ml penicillin and 100 g/ml streptomycin, and cut into small pieces. The minced amnion was digested with 0.25% trypsin (Sigma-Aldrich Co., Steinheim, Germany) and collagenase I (0.75 mg/ml) in Dulbecco's Modified Eagle's Medium (DMEM; Sigma, Irvine, UK) for 70 min at 37°C. The harvested cells were cultured in DMEM complete medium composed of DMEM medium supplemented with 10% heat-inactivated fetal bovine serum (FBS), 100 U/ml penicillin, 100 g/ml streptomycin and 2 mM L-glutamine (sigma-Aldrich) at 37°C in a humidified atmosphere with 5% CO_2_. The passages 3^rd^ to 7^th^ cells were used in this study. To estimate their potential to differentiate into several tissue lineages, the cells from the amnion were cultured in adipogenic and osteogenic medium, respectively.

### Mouse liver cirrhosis model generation and hAMCs transplantation

Liver fibrosis was induced with CCl_4_ according to the previous report [22]. Mice were injected intraperitoneally (IP) with CCl_4_ (50% in olive oil, 1 ml/kg, twice a week) for 8 weeks. After final CCl_4_ injection at 4 weeks, animals were randomly divided into two groups: the hAMCs group and saline group (10 mice each group). One day (24 hours) after the eighth injection of CCl_4_, 1×10^5^ hAMCs or same volume of saline as a control were injected into the spleen under anesthesia with diethyl ether as described previously [23], [24]. No immunosuppressive reagent was given after the cell transplantation.

At the time of sacrifice, blood was obtained from the aorta for measurement of liver functions, and the samples were stored at −20°C.

The liver was removed, rinsed with PBS, and divided into four portions: (a) fixed in 10% buffered formaldehyde formalin and embedded in paraffin; (b) snap frozen at −70°C for sectioning and immunohistochemistry; (c) homogenized in appropriate buffer(s) and aliquots frozen at −70°C for biochemical assays; and (d) snap frozen at −70°C for DNA isolation.

### Assessment of liver functions

Elevations in serum alanine aminotransferase (ALT) and aspartate aminotransferase (AST) generally reflect hepatocyte injury, which were measured by automated biochemical analyzer.

### Liver histology and morphometric collagen determination

The liver paraffin sections (5 µm) were stained with H&E for histological analysis or Masson trichrome for collagen deposition. The percentage of fibrotic area in the liver was measured with a computer-assisted automated image analyzer Image-Pro Plus 5.1 (Media Cybernetics Inc., Bethesda, MD, USA). This analyzer measured ten random fields per slide and calculated the ratio of connective tissue to the whole area of the liver.

### Quantitation of stellate cells

The activated HSC is a key cell type that contributes to liver fibrosis [6]. Upon liver damage, HSC become activated and produce excessive extracellular matrix, leading eventually cirrhosis and liver failure. To observe whether transplanted hAMCs could inhibit the activation of HSC, the expression of desmin, alpha-smooth muscle actin (α-SMA) and glial fibrillary acidic protein (GFAP) were examined by immunofluorescent staining in deparaffinized liver sections. The sections were incubated with primary antibody at 4°C overnight. After the sections were washed with PBS, they were incubated with secondary antibody, FITC-labeled goat anti-mouse immunoglobulin G (1∶200 dilution; BD Pharmingen), or Rodamin-labeled goat anti-mouse immunoglobulin G (1∶200 dilution; Dako). After they had been washed in PBS, the sections were mounted with a medium containing 4-,6-diamidino-2-phenylindole (DAPI) as a counter stain (1∶200 dilution, Dako), coverslipped, and examined under a fluorescence microscope. Cells were counted in the eight fields per slide for each mouse (magnification ×200).

### Measurements of hepatocyte regeneration and apoptosis

To evaluate hepatocyte regeneration, deparaffinized liver sections were incubated overnight at 4°C with mouse monoclonal antibodies against proliferating cell nuclear antigen (PCNA, 1∶200 dilution, BD Pharmingen).

To evaluate hepatocyte apoptosis, the terminal deoxynucleotidy transferase-mediated dUTP nick end-labeling (TUNEL) assay was performed using an In Situ Cell Death Detection Kit (Roche, Germany) according to the manufacturer's instructions.

The PCNA and apoptotic indices were defined by calculating the number of positive cells per field at ×200 magnification in each complete liver section.

### Measurement of senescence-associated β-gal activity

Detection of Senescence-associated β-Gal staining (SA-β-Gal) was performed as described previously [25]. SA-β-Gal -positive were quantified by counting stained and unstained cells and expressed as the percentage of SA-β-Gal -positive cells over the total counted cells.

### Detection of human cells by DNA-PCR

To determine if hAMCs were present in murine organs, DNA was extracted from liver using phenol chloroform and amplified by PCR using primers specific for human Alu repeat sequences. DNA extracted from healthy mouse liver served as a control.

### Measurements of antioxidant activity

The enzymatic activities of superoxide dismutase (SOD) were measured through xanthine-oxidation method according to the instructions (Jiancheng Institute, Nanjing, China) as described previously [26].

### Monitoring of implanted hAMCs in injured liver

Additional mouse were used to examine whether transplanted hAMCs differentiate into hepatocyte in the injured liver. Cells were labeled with DiI (Molecular Probes Inc., Eugene, USA) for cell tracking [27], [28] according to the manufacturer's protocol before the transplantation. Animals were killed 4 weeks after transplantation. Liver were fixed in 4% paraformaldehyde and processed for cryosection. Potential transformation to hepatocyte cells from engrafted hAMCs was verified by antibody immunostaining for human albium protein (1∶200 dilution, Abcam) and α-fetoprotein (1∶200 dilution, Abcam). FITC-conjugated IgG antibody (1∶400 dilution, BD Pharmingen) was used as a secondary antibody. All morphometric studies were performed using a confocal microscope by two experienced technicians blinded to the treatment.

### Western blot analysis

Liver sections were lysed in RIPA buffer (50 mM Tris–HCl, pH 7.4, 150 mM NaCl, 1.0% Triton X-100, 0.5% sodium deoxycholate, 1.0 mM sodium orthovanadate, 0.1% sodium dodecyl sulfate (SDS), 1.0 mM phenylmethyl-sulfonylfluoride, 1.0 mg/ml aprotinin, 10 mg/ml leupeptin, and 1.0 mg/ml pepstatin A in autoclaved ddH2O) for 30 min on ice. Lysates were centrifuged (13,000 g, 15 min, 4°C) and supernatants were collected. Protein lysates (30 µg) were electrophoresed on sodium dodecyl sulfate-polyacrylamide gels and transferred onto nitrocellulose membranes. After blocking, membranes were incubated overnight at 4°C with specific antibodies against hepatocyte growth factor (HGF), p16^INK4a^, p21^Cip1^, p27^Kkip1^, Sirt-1, Prdx-1 and bcl-2 (All from santa cruz). After three 5 min washings, the membranes were incubated with horseradish peroxidase-conjugated secondary antibody, washed and visualized with an ECL detection kit (Amersham Pharmacia, New Jersey, USA).

### Statistical analysis

Data are expressed as mean ± SEM. Statistical analysis was performed using independent sample-*t* test. A p-value of <0.05 was taken to indicate statistical significance.

## Results

### hAMCs characterization

**Figure 1:**
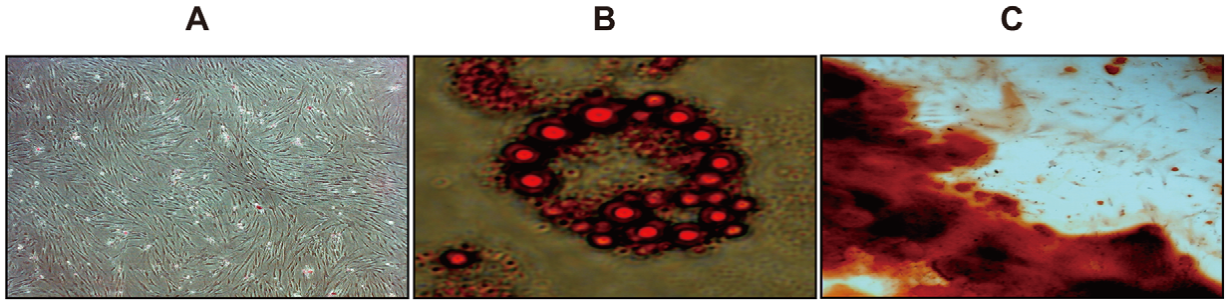
Characterization of human amniotic membrane-derived mesenchymal stem cells. (A) Human amniotic membrane-derived mesenchymal stem cells (hAMCs) display fibroblastic morphology in cultures. (B) and (C) The differentiation of hAMCs into adipocytos and osteoblasts in cultures. Cells were incubated in adipogenic or osteogenic medium and then analyzed by cytochemical staining with Oil Red-O (B) and Alizarin red (C), respectively.

During the primary cell cultures, the attached cells stretched and took the shape of a typical spindle-shaped fibroblast phenotype (Fig. 1A). These adherent cells could be readily expanded in vitro by successive cycles of trypsinization, seeding and culture every 3 days for 15 passages without visible morphologic change. At the end of the induction period, the cells differentiated into adipogenic and osteogenic lineages (Figs. 1B and C).

### hAMCs transplantation ameliorated the CCl_4_-induced deterioration of liver function

Mice treated with CCl_4_ alone had 4-fold increase of serum ALT (539.6±545.1 U/dl) and 2.5-fold increase of serum AST (589.7±342.8 U/dl) activity relative to those with hAMCs group (ALT 139.1±138.3 U/dl, p<0.05 and AST 212.3±110.7 U/dl, p<0.01), respectively. These results suggested that hAMCs transplantation ameliorates the CCl_4_-induced deterioration of liver function.

### hAMCs transplantation alleviated CCl_4_-induced liver fibrosis

**Figure 2:**
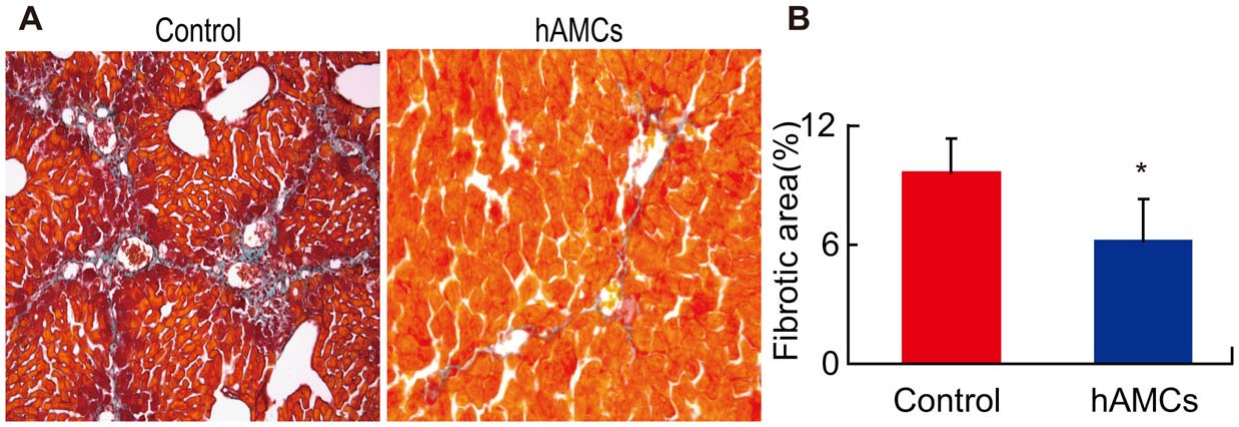
hAMCs transplantation reduced hepatic fibrosis. (A) Micrographs of Masson's trichrome stained liver paraffin sections from control group and hAMCs group. (B) Fibrotic areas were quantified by computer assisted image analysis. Each value is the mean±SEM of determinations in 8 mice of each group. *p<0.05 compared with control group.

After 8 weeks of CCl_4_ challenge, liver fibrosis was observed in Masson-stained sections. hAMC transplantation had 1.5-fold decrease of fibrotic area (6.2%±2.1 vs. control 9.6%±1.7, p<0.05) and made the thickened septal fibrosis thinning or disappears (Fig. 2). Thus, hAMC transplantation prevented and ameliorated the formation of liver fibrosis.

### hAMCs transplantation reduced CCl_4_-induced hepatic stellate cell activation

**Figure 3:**
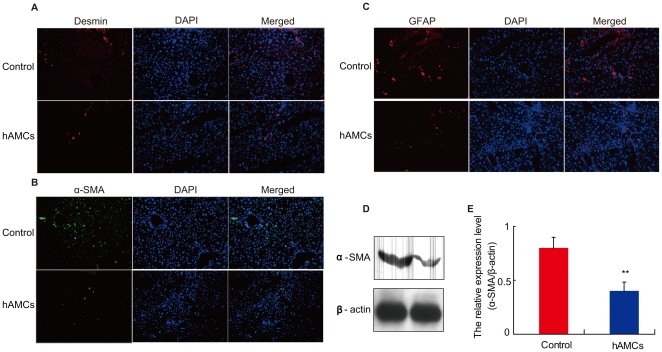
hAMCs transplantation reduced CCl_4_-induced hepatic stellate cells activation. (A) Micrographs of liver frozen sections from control group and hAMCs group stained with immunofluorescence for Desmin (left panel), DAPI (middle panel) and merged (right panel). (B) Micrographs of liver frozen sections from control group and hAMCs group stained with immunofluorescence forα-SMA (left panel), DAPI (middle panel) and merged (right panel). (C) Micrographs of liver frozen sections from control group and hAMCs group stained with immunofluorescence for GFAP (left panel), DAPI (middle panel) and merged (right panel). (D) Representative Western blots of liver extracts from control group and hAMCs group for expression of α-SMA. β-actin was used as an invariant control. (E) α-SMA protein levels relative to β-actin protein level were assessed by densitometric analysis. Each value is the mean±SEM of determinations in 5 mice of each group. ** p<0.01 compared with control group.

After 8 weeks of CCl_4_ challenge, desmin, α-SMA- and GFAP-positive HSCs were strongly and diffusely present near the expanding septa and in the perisinusoidal spaces of sidual hepatic parenchyma. hAMCs transplantation significantly reduced the percentages of these positive HSCs [desmin 35.2%±5.8, α-SMA 52.3%±11.2, GFAP 51.7%±12.2 in control group vs. desmin 17.2%±3.8, α-SMA 22.3%±8.5, GFAP 21.1%±9.2 in hAMCs group, p<0.05, Fig. 3]. Meanwhile, the expression of α-SMA protein was obviously lower in hAMCs group compared with control group (p<0.01). Thus, hAMCs transplantation prevented and reduced the HSC activation.

### hAMCs transplantation suppressed hepatocyte apoptosis and promoted hepatocyte regeneration

**Figure 4:**
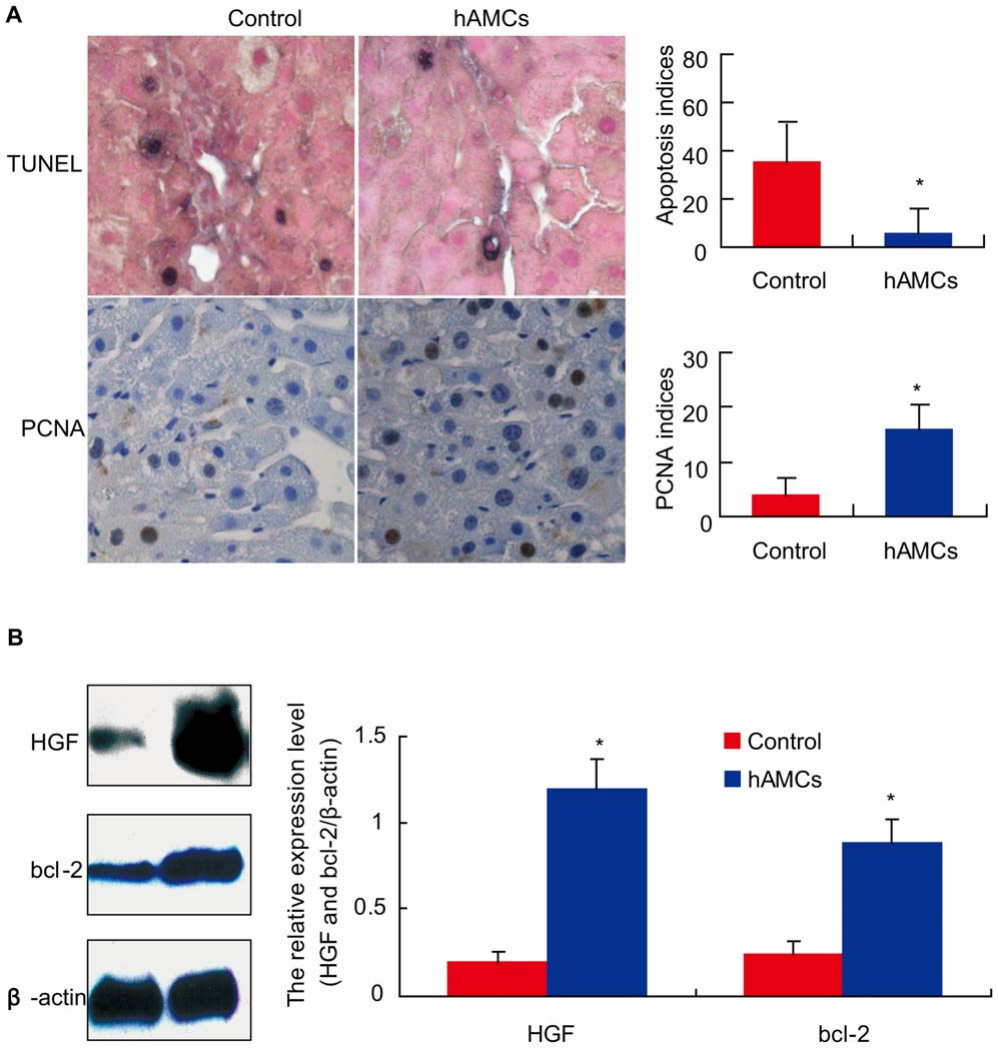
hAMCs transplantation suppressed hepatocyte apoptosis and promoted hepatocyte regeneration. (A) Micrographs of liver paraffin sections from control group and hAMCs group stained immunohistochemically for TUNEL and PCNA. (B) Representative Western blots of liver extracts from two groups for expression of HGF and bcl-2. β-actin was used as an invariant control. HGF and bcl-2 protein levels relative to β-actin protein level were assessed by densitometric analysis. Each value is the mean±SEM of determinations in 8 mice of each group. *p<0.05 compared with control group.

To observe whether the reduction of serum ALT and AST were associated with reduced hepatocyte apoptosis, TUNEL assay was performed. Quantification revealed a 70% reduction in TUNUL-positive hepatocyte-nuclei in hAMCs group (5.2±11/field of view) compared with controls (35.2±32/field of view; p<0.05, Fig. 4). We also investigated whether hAMCs transplantation induced hepatocyte proliferation. As shown in Fig. 4, few PCNA-positive hepatocytes were detected in control livers, however, many were observed in hAMCs group. The number of proliferating liver cells were increased 4-fold in hAMCs group (16±4.3/field of view) compared with control group (3.8±3.2/field of view; p<0.05).

HGF can stimulate hepatocyte to replicate and inhibit apoptosis and the upregulation of bcl-2 is also associated with decrease of cellular apoptosis. As shown in Fig. 4, the HGF and bcl-2 protein levels in injured livers were upregulated significantly in hAMCs group compared with control group. Thus, hAMCs transplantation prevented hepatocyte apoptosis and ameliorated hepatocyte proliferation.

### hAMCs transplantation suppressed hepatocyte senescence

**Figure 5:**
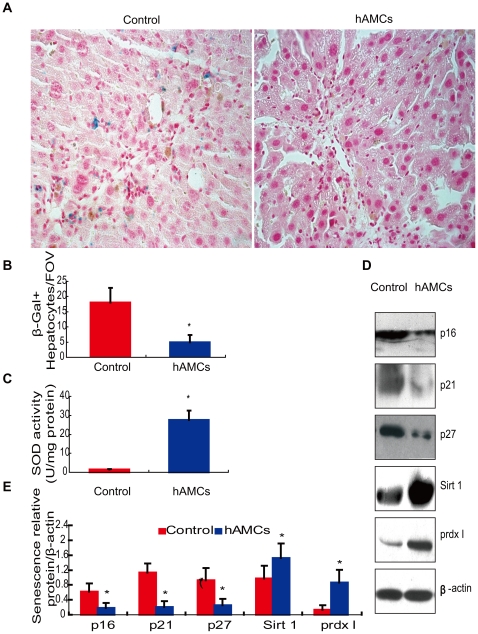
hAMCs transplantation suppressed hepatocyte senescence. (A) Micrographs of liver section stained histochemically for SA-β-Gal. (B) SA-β-Gal positive hepatocytes per field. (C) The levels of SOD activity in mouse liver homogenate from control group and hAMCs group. (D)Representative Western blots of liver extracts from control group and hAMCs group for expression of p16^INK4a^, p21^Cipl^, p27^Kipl^, Sirt 1 and prdx I expression in livers from hAMCs group or control group. β-actin was used as an invariant control. (E) p16^INK4a^, p21^Cipl^, p27^Kipl^, Sirt 1 and prdx I protein levels relative to β-actin protein level were assessed by densitometric analysis. Each value is the mean±SEM of determinations in 8 mice of each group. FOV = Field of view. *p<0.05 compared with control group.

To test whether the ameliorated effect of hAMCs transplantation on the CCl_4_-induced liver fibrosis was associated with decreasing hepatocyte senescence, senescence-associated β-Gal activity was examined. As shown in Fig. 5, intense staining was observed in the livers from control group, however, senescence-associated β-Gal activity was seldom detected in the livers from hAMC group.

The expression of senescence-relative proteins, including p16^INK4a^, p21^Cip1^, p27^Kkip1^ and Sirt-1 were also examined in livers. Results showed that the expression of p16^INK4a^, p21^Cipl^ and p27^Kipl^ was down-regulated significantly, whereas the expression of Sirt-1 was up-regulated significantly in hAMCs group compared with control group (Fig. 5).

To observe whether the anti-senescence role of hAMCs was associated with enhanced antioxidant activity, the levels of SOD activity in mouse liver homogenate were analyzed. The results showed that the levels of SOD activity were raised significantly in hAMCs group compared with control group (Fig. 5). The protein expression of Prdx I, a member of the peroxiredoxin family was also examined and found that it was upregulated in hAMC group compared with in control group (Fig. 5). Thus, hAMC transplantation prevented and ameliorated the hepatocyte senescence.

### hAMCs engraft in CCl_4_ injured liver

**Figure 6:**
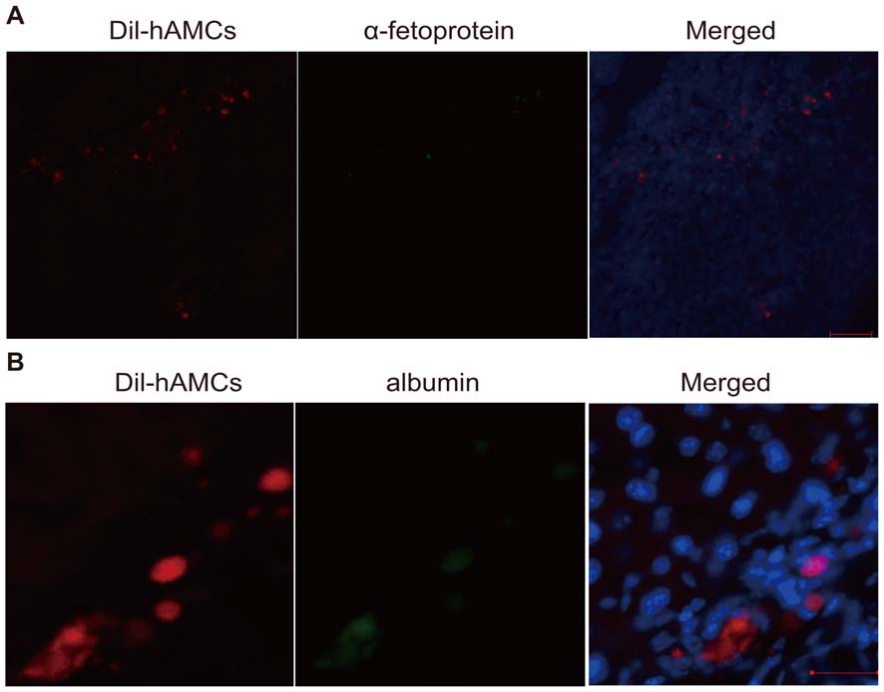
hAMCs engraft in CCl_4_ injured livers. (A) Micrographs of liver frozen sections stained with immunofluorescence for DiI (left panel), α-fetoprotein (middle panel) and merged (right panel). (B) Micrographs of liver frozen sections stained with immunofluorescence for DiI (left panel), albumin (middle panel) and merged (right panel). Bars = 100 µm.

Four weeks after hAMC transplantation, DiI-labeled cells were observed around the portal tracts (Fig. 6), in the fibrotic areas and around inflammatory sites of liver. This result suggests that the transplanted cells migrated through the blood circulation from the spleen into the sinusoid and liver parenchymal tissue.

To elucidate the mechanism of the cell therapy by confirming and monitoring the hepatic differentiation of hAMCs, human-specific antibodies with no cross-reactivity to mouse antigens were used to label the liver-associated markers human albumin and α-fetoprotein. Four weeks following cell transplantation, the transplanted DiI-labeled hAMCs were detected and expressed human α-fetoprotein and albumin in mouse liver sections (Fig. 6).

DNA-PCR for human Alu repeat sequences confirmed the presence of human cells in the liver of CCl_4_ treated mice. Thus the transplanted hAMCs could differentiate into albumin-secreting hepatocyte-like cells in the damaged liver.

## Discussion

The aim of this study was to evaluate the effect of hAMCs transplantation for the improvement of hepatic fibrosis. The major findings in our study were as follows: (1) the administration of hAMCs into CCl_4_-induced fibrosis mouse by the spleen delayed disease progression and recovered liver function; (2) hAMCs transplantation inhibited HSC activation; (3) hAMCs transplantation reduced hepatocyte apoptosis and promoted hepatocyte proliferation; (4) hAMCs transplantation depressed hepatocyte senescence; and (5) transplanted hAMCs migrated into injured liver and expressed hAlb and hAFP.

HSCs are the predominant source of extracellular matrix collagen in the liver, playing a crucial role in the pathogenesis of hepatic fibrosis [6]. HSCs activation is the earliest step in hepatic fibrogenesis, collagen deposition, and progressive fibrosis leading to cirrhosis. When activated, HSCs transform into myofibroblast-like cells. Since the first report by Yokoi et al [29], desmin has frequently been used as a marker of HSCs and reflecting the proliferation kinetics of HSCs after CCl_4_ intoxication or bile duct ligation. In addition, amount of evidences demonstrated that the expression of a-SMA is a reliable and widely used marker of activation of HSCs [30], [31]. GFAP, firstly considered to be specific for cells of astroglial lineage, is an alternative indicator for the activation of HSCs and consequent liver fibrogenesis [32], [33]. In this study, we investigated the effect of hAMCs transplantation on HSCs activation in CCl_4_-induced cirrhotic mouse. Our study showed that hAMCs decreased HSCs activation, as indicated by decreasing expression of a-SMA, GFAP and desmin.

In response to CCl_4_ treatment, hepatocyte may undergo apoptosis in addition to necrosis [34]. In our mouse model under chronic CCl_4_ administration, hepatocyte apoptosis was suppressed with increased anti-apoptotic protein bcl-2 and HGF expression in the livers by hAMCs transplantation. Apoptosis of hepatocytes is one of the major promoting factors in the development of liver fibrosis [35]. Previous study [36] has demonstrated that DNA from apoptotic hepatocytes acts as an important mediator of HSCs differentiation and inducing HSCs collagen production, thus promotes liver fibrogenesis. In this study, significantly suppressed HSCs activation and less collagen accumulation in hAMC transplanted mice were observed, which might be contributed in part to the reduced hepatocyte apoptosis.

Hepatic regeneration is an important component of the recovery process occurred after liver injury, and the improvement of hepatocyte proliferating capacity could be of critical importance in CCl_4_-induced cirrhosis. In liver regeneration, the existence of liver specific growth factors has been extensively studied. HGF, as the most effective mitogen, inhibits liver injury by stimulating hepatocyte proliferation in addition to protecting hepatocyte from apoptosis [37], [38]. In the present study, we confirmed the hAMCs transplantation induced higher expression of HGF protein in CCl_4_-induced liver injury. In additional to a decreased hepatocyte apoptosis, we also found a more significant hepatocyte proliferation in hAMC group relative to control group. Therefore, the improvement of liver functions by the hAMCs transplantation was partially contributed to enhancing hepatocyte proliferation.

One of the unique responses of the injured liver is hepatocyte replication and regeneration. However, somatic cells have a limited capacity for replication owing to lack of the telomerase enzyme. Increased hepatocyte senescence has been confirmed in cirrhosis [39] and has been shown to correlate with the ductular reaction and portal fibrosis in chronic hepatitis C and with clinical severity [40]. So far, SA-β-gal activity has been used as a marker of cellular senescence [25], [41]. In this study, profound hepatocyte senescence was confirmed after CCl_4_-induce liver injury as SA-β-gal activity was detected frequently in hepatocyte in CCl_4_-treated liver. Whereas, hAMCs transplantation depressed cellular senescence in livers. To the best knowledge of our known, this is the first report that hAMCs transplantation decreased CCl_4_-induce cellular senescence. Evidences strongly support that increased expression of p16 ^INK4a^ and p21^Cip1^ are the key steps in the development of cellular senescence [42], [43]. Moreover, the Cdk inhibitor p27^Kip1^, which is involved in several forms of G1check point control, was also correlated with increased cellular senescence. In consistent with decrease SA-β-gal activity, the expression of senescence-relative proteins of p16 ^INK4a^, p21^Cip1^ and p27^Kip1^ were down-regulated significantly, whereas the expression of Sirt1 was upregulated significantly in hAMCs group relative to control group. NAD-dependent deacetylase Sirt protein play an important role in the survival of cell. The upregulation of Sirt1 could promote cellular proliferation, reducing senescence and apoptosis [44], [45], [46]. In addition, oxidant stress has been demonstrated to inducing cellular senescence and apoptosis [47]. In the present study, SOD activity, an anti-oxidant enzyme, was noticeable higher in hAMCs group than control group. Our results also found that the expression of Prdx 1, a member of the peroxiredoxin family of antioxidant enzymes, was upregulated significantly in hAMC group than control group. Taken together, it seems that hAMCs transplantation decreased CCl_4_-induced hepatocyte senescence by depressing oxidant stress, upregulating Sirt 1 expression and reducing p16 ^INK4a^, p21^Cip1^ and p27^Kip1^ expression.

Previous studies have demonstrated that hAMCs can differentiate into hepatocyte lineage cells in vitro [20], [48]. In our study, hAMCs were successfully transplanted via spleen into hepatic cirrhosis model to prevent histopathological changes. These cells survived and scattered in the liver at 4 weeks after the transplantation. Moreover, they also differentiate into albumin-expressing or a-fetoprotein–expressing hepatocyte. Therefore, the effect of hAMCs on reducing fibrogenesis partially likely relies on the differentiation of these cells into hepatocytes in vivo.

In summary, these results suggest that hAMCs transplantation significantly decrease the fibrosis formation and progression of CCl_4_-induced cirrhosis, providing a new approach for the treatment of fibrotic liver disease.
